# Multi-timescale Modeling of Activity-Dependent Metabolic Coupling in the Neuron-Glia-Vasculature Ensemble

**DOI:** 10.1371/journal.pcbi.1004036

**Published:** 2015-02-26

**Authors:** Renaud Jolivet, Jay S. Coggan, Igor Allaman, Pierre J. Magistretti

**Affiliations:** 1 Department of Neuroscience, Physiology & Pharmacology, University College London, London, United Kingdom; 2 Brain Mind Institute, École Polytechnique Fédérale de Lausanne (EPFL), Lausanne, Switzerland; 3 NeuroLinx Research Institute, La Jolla, California, United States of America; 4 Biological and Environmental Sciences and Engineering Division, King Abdullah University of Science and Technology (KAUST), Thuwal, Kingdom of Saudi Arabia; UFR Biomédicale de l’Université René Descart, France

## Abstract

Glucose is the main energy substrate in the adult brain under normal conditions. Accumulating evidence, however, indicates that lactate produced in astrocytes (a type of glial cell) can also fuel neuronal activity. The quantitative aspects of this so-called astrocyte-neuron lactate shuttle (ANLS) are still debated. To address this question, we developed a detailed biophysical model of the brain’s metabolic interactions. Our model integrates three modeling approaches, the Buxton-Wang model of vascular dynamics, the Hodgkin-Huxley formulation of neuronal membrane excitability and a biophysical model of metabolic pathways. This approach provides a template for large-scale simulations of the neuron-glia-vasculature (NGV) ensemble, and for the first time integrates the respective timescales at which energy metabolism and neuronal excitability occur. The model is constrained by relative neuronal and astrocytic oxygen and glucose utilization, by the concentration of metabolites at rest and by the temporal dynamics of NADH upon activation. These constraints produced four observations. First, a transfer of lactate from astrocytes to neurons emerged in response to activity. Second, constrained by activity-dependent NADH transients, neuronal oxidative metabolism increased first upon activation with a subsequent delayed astrocytic glycolysis increase. Third, the model correctly predicted the dynamics of extracellular lactate and oxygen as observed *in vivo* in rats. Fourth, the model correctly predicted the temporal dynamics of tissue lactate, of tissue glucose and oxygen consumption, and of the BOLD signal as reported in human studies. These findings not only support the ANLS hypothesis but also provide a quantitative mathematical description of the metabolic activation in neurons and glial cells, as well as of the macroscopic measurements obtained during brain imaging.

## Author Summary

The brain has remarkable information processing capacity, yet is also very energy efficient. How this metabolic efficiency is achieved given the spatial and metabolic constraints inherent to the designs and energy requirements of brain cells is a fundamental question in neurobiology. The major cell classes in mammalian nervous systems include neurons, glia and the microvasculature that supplies the molecular substrates of energy and metabolism. Together, this neuron-glia-vasculature (NGV) ensemble constitutes the functional unit that underlies the cost infrastructure of computation. In spite of its importance, a comprehensive understanding of this dynamic system remains elusive. While it is well established that glucose feeds the brain, few of the details regarding the destiny of glucose intermediates in metabolic pathways are known. Controversy remains regarding the degree of cooperativity between glia and neurons in sharing lactate, the product of aerobic glycolysis (Warburg effect) and one of the substrates for further energy extraction by oxidative processes. Specifically, while experimental data support the occurrence of a flow of lactate from glia to neurons, the astrocyte-neuron lactate shuttle (ANLS), some theoretical considerations have been proposed to support the occurrence of lactate transport in the other direction (NALS). Our computational model is the first to integrate multiple timescales of the NGV unit. It provides a quantitative mathematical description of metabolic activation in neurons and astrocytes, and of the macroscopic measurements obtained during brain imaging that uses metabolism as a proxy for neuronal activity.

## Introduction

The mammalian brain exhibits remarkable processing power. It is at the same time energy efficient. The design features that allow such efficient computation are mapped in cellular and molecular components and their roles in information processing. Concurrently, these features are anchored in, and constrained by, the universal metabolic chains that provide energy to cells. Deciphering the metabolic code and the neural code are thus tandem requirements for a comprehensive understanding of brain function. Understanding the metabolic underpinnings of information processing is also of added value to understanding the etiology and progression of neuropsychiatric and neurodegenerative disorders [1, 2]. The picture that emerges from this dynamical system will reflect the cooperative function of neurons, glia and the vascular system.

Glutamate, the brain’s major neurotransmitter, effects numerous cascades and processes in brain cells [3, 4]. Among them, astrocytes couple synaptic activity to energy metabolism via a sodium-dependent uptake of glutamate [5]. The ensuing cascade of molecular events leads to the glycolytic processing of glucose and the release of lactate by astrocytes. A comprehensive model of brain energy metabolism must consider oxidative and non-oxidative glucose consumption, intracellular and extracellular compartmentalization and transport of choke-point metabolic intermediates such as lactate and pyruvate, as well as feedback mechanisms that report local synaptic and intrinsic neuronal activity [6, 7]. These pathways are in turn complicit in the molecular and cellular mechanisms that contribute to the still poorly understood read-out of functional brain imaging [8].

The role of astrocytes and how they metabolically interact with neurons is well supported experimentally; some mostly theoretical considerations, however, have challenged this view. Magistretti and colleagues proposed that clearance of glutamate from the synaptic cleft by astrocytes could be coupled to glycolysis and subsequent lactate production [5]. Lactate produced in this way would then be transported to the extracellular space. Controversy remains surrounding directionality and timing of lactate flow in the brain; while a neuron-to-astrocyte lactate system (NALS) is proposed by some [9, 10], an astrocyte-to-neuron direction (ANLS) is supported by a large set of experimental evidence [11]. Biophysical models also weigh-in on the conditions and sequences of events required for lactate production and consumption [12–15].

The existence of an extracellular pool of lactate likely used as an energy reservoir at the onset of stimulation has been observed in rats and humans [16, 17]. The distribution of monocarboxylate transporters at the membrane of neurons and astrocytes supports the hypothesis of a net transfer of lactate from astrocytes to neurons through the extracellular space [18]. Glial cells have been observed to take up most glucose [19, 20], while neurons are responsible for the largest part of brain oxygen consumption [21, 22]. Additional evidence comes from the direct measure of NADH transients in brain slices, showing that neurons display early oxidative metabolism following presynaptic activity, while astrocytes display a delayed activation of glycolysis but no detectable oxidative response [23]. Nevertheless, the ANLS-hypothesis is still debated and challenged with arguments focusing now on the exact interpretation of the above observations [24, 25].

Nicotine adenine dinucleotide, either oxidized or reduced (NAD^+^, or NADH), is a workhorse cofactor that acts as a central electron broker for metabolic redox cycles including glycolysis, the citric acid cycle (Krebs, TCA) and oxidative phosphorylation. Owing to its high UV wavelength absorption, it is also responsible for cellular auto-florescence. This coincidence makes it a useful indicator of metabolic activity. NADH is an important metabolic signal because it is produced or used during both mitochondrial activity and activation of the glycolytic pathway, and because it cannot diffuse freely through the mitochondrial membrane but needs to be transported by appropriate shuttles. Fluctuations of the NADH concentration measured in the appropriate cellular compartments can then indicate increased or decreased oxidative and glycolytic metabolism. A critical previous finding in this regard was the observation of early and late activity-dependent phases of metabolic activity with the early phase taking the form of a NADH “dip” and the late phase appearing as a NADH “overshoot” with a longer time constant of decay [23]. Interestingly, these phases also correlate with the fluctuations of the extracellular lactate concentration as determined in animals [16] and humans [26, 27].

The emerging consensus is that the early phase represents NADH depletion in the dendrites of active neurons and that the overshoot represents glycolytic activity that results in the accumulation of NADH. This activity results in the high production of lactate in astrocytes as rapid glycolysis overtakes the subsequent consumption by oxidative pathways [23, 26, 27]. The accumulation and transportation of lactate between glial cells and neurons may in turn serve as an activity-dependent buffer that is informed by the neuronal release and glial uptake of glutamate [5]. It might also act as a signaling molecule to the vasculature [28] or to brain cells via binding to the G-protein coupled receptor GPR81 [29].

The preference for lactate over glucose as an energy substrate in neurons has been demonstrated *in vivo* as well as *in vitro* [30, 31], as has a neuroprotective role for lactate in the case of insulin-dependent hypoglycemia [32] and other conditions [33, 34]. The role of the ANLS in homeostatic maintenance involves the regulation of blood glucose [35] and sodium [36]. While the ANLS hypothesis, since its initial formulation [5], does not preclude the use of glucose by neurons as an energy substrate, it has been challenged by some studies defending the view that glucose, rather than lactate, is the sole energy substrate for oxidative metabolism in neurons [37, 38].

Previous modeling efforts have advanced our knowledge of this functional metabolic network by demonstrating that lactate consumption by neurons occurs early in the stimulus regimen and that the early and late lactate transients correspond to the activity of two distinct populations of cells, neurons and glia. The current study builds on and complements those and other models [9, 10, 12–15, 39–42] and addresses unresolved mechanisms of neuron-glial metabolic and vascular coupling. The model is based on several previous studies [13, 40] with five significant improvements: 1) the compartmentalization of NADH between cytosolic and mitochondrial compartments; 2) the linking of metabolic and Hodgkin-Huxley formalisms; 3) the input to the neuronal and astrocytic compartments formulated as a presynaptic glutamatergic stimulation; 4) the model explicitly and continuously updates reversal potentials; and 5) the model was constrained using *in vitro* data and correctly predicts *in vivo* results without the need of invoking glycogen (which is deliberately excluded from the model).

In this paper, we will show that a biophysical model of astrocyte-neuron metabolic interactions designed following these principles leads to the presence of an activity-dependent lactate shuttle from astrocytes to neurons and that this model can reproduce the evoked response of NADH in its various compartments as reported by Kasischke and colleagues [23]. We will subsequently show that our biophysical model correctly predicts qualitatively—and to some extent quantitatively—the evoked responses of tissue lactate and tissue oxygen as observed in the rat brain *in vivo* [16]. Finally, our biophysical model predicts the evoked responses of tissue lactate, of the BOLD signal and the glucose and oxygen consumption as observed in the human brain *in vivo*.

## Methods

**Figure 1:**
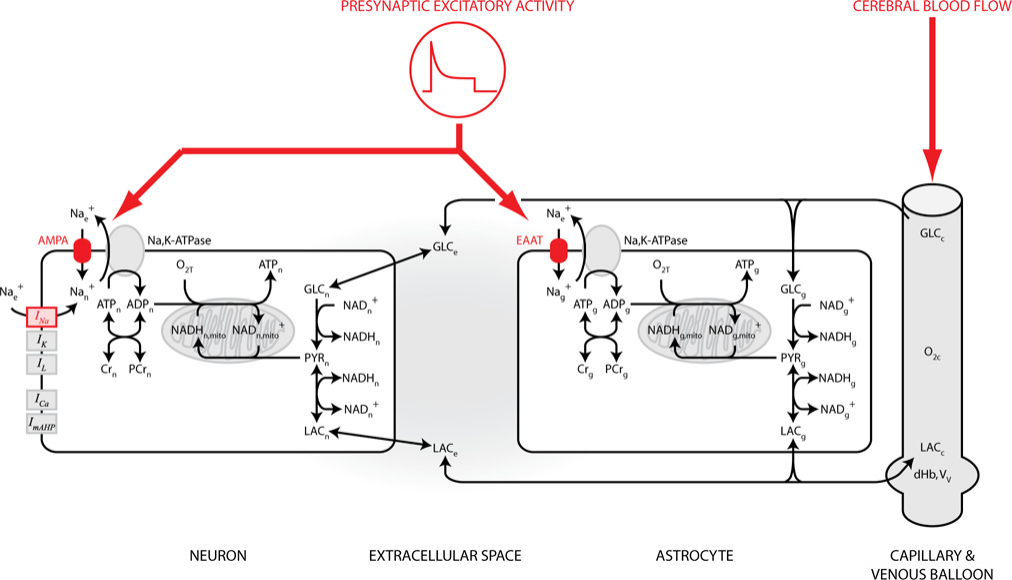
Model structure. The model is divided in four main compartments: a neuronal compartment, an astrocytic compartment, the extracellular space and a vascular compartment. Neurons and astrocytes are further divided between a cytosolic sub-compartment and a mitochondrial sub-compartment to account for the compartmentalization of oxidative and glycolytic metabolisms. Both neurons and astrocytes contain a metabolic network including glycolytic enzymes, lactate dehydrogenase, glucose and lactate transporters, NADH shuttles, oxidative metabolism, phosphocreatine and the Na, K-ATPase electrogenic pump. Additionally, the neuronal compartment contains voltage- and calcium-gated ion channels following the Hodgkin-Huxley formalism. The system as a whole is driven by two independent inputs (red). First, a glutamatergic presynaptic population activates AMPA receptors on the neuronal membrane and excitatory amino-acid transporters (EAATs) on the astrocytic membrane. The activation of both AMPA receptors and EAATs leads to an increase of the intracellular sodium concentration which activates the energy consuming Na, K-ATPase pump and subsequent metabolic processes. Activation of AMPA receptors also depolarizes neurons and might lead to the generation of action potentials, which will also lead to an increase in intracellular sodium in the neuronal compartment via opening of voltage-gated sodium channels. Second, the cerebral blood flow (CBF) is modulated as a separate input. For comparison to *in vitro* experiments using acute brain slices, the only input is the presynaptic neuronal population while the supply of oxygen and glucose that normally comes from the CBF is held constant as a proxy for the laminar flow of a controlled perfusing solution. Finally, the extracellular space is the place where cells and capillaries exchange metabolites

This *in silico* model represents a dynamic and integrative analysis of compartmentalized metabolism and its relation to neuronal signaling in the central nervous system. The model was designed based on knowledge of the underlying biophysics and required input parameters and equations from multiple species, time and spatial scales. The model is inspired by previous work from Aubert and colleagues [13, 40] and consists of four compartments: neuron, astrocyte, capillary and extracellular space (see Fig. 1). These compartments are referred to by the subscripts *n*, *g* (for glia/astrocyte), *c* and *e* respectively. In addition, the neuronal and astrocytic compartments are further divided between cytosolic and mitochondrial sub-compartments to account for the compartmentalization of nicotinamide adenine dinucleotide (NADH). These are referred to by the superscripts *cyto* and *mito*. Transport between compartments is noted with the subscripts of both compartments; for instance, transport from the neuronal compartment to the extracellular space is labeled with *ne* or, conversely, *en*.

The model is formulated as a series of 33 differential equations adapted from previous work [13, 40] with the following improvements: the compartmentalization of (mostly) NADH between the cytosolic and mitochondrial compartments; the model was joined to a Hodgkin-Huxley-type model [43, 44]; the input to the neuronal and astrocytic compartments is formulated as a glutamatergic input and not anymore as an abstract stimulation; the model explicitly models sodium entry and extrusion in both the neuronal and astrocytic compartments, continuously updating the corresponding reversal potentials. The model, which for the first time bridges mathematical descriptions of energy metabolism and Hodgkin-Huxley equations, was constrained on *in vitro* data and correctly predicts *in vivo* results.

Parameters that were difficult to determine experimentally such as transport constants were left free to vary [10, 42]. Free parameters were then optimized so that the model reproduces the experimental results presented in [23]. The number of free parameters was maintained as small as possible by enforcing constraints on the value of metabolites at steady state. Simulations with randomized fluctuations in parameter values (up to plus or minus 10% of reported values) did not reveal significant changes in behavior of the model. This approach successfully predicts qualitatively and quantitatively *in vivo* measurements in rodents and in humans (see Results, Fig. 5 and 6).

The pooling together of equations from various sources is unfortunately necessary for construction of such a broad and multi-dimensional model. There is no single source of equations that can be tapped for this model. Note however that most equations come from only two published sources. The variables, their steady-state values and the corresponding governing equations are given in Table 1. Equations (A.1)-(A.8), (A.13) and (A.16)-(A.22) are taken from Aubert and Costalat [40] and were originally introduced in ref. [45] for the equations describing metabolism and by Buxton and colleagues [46] for the equations describing the vascular dynamics. Equations (A.9)-(A.12), (A.14) and (A.15) are original. Equations (A.23)-(A.26) describe the neuronal membrane excitability following the Hodgkin-Huxley formalism and the neuronal calcium dynamics. They are adapted from [44].

**Table 1:** Governing equations.

**Variable**	**Value at rest**		**Equation**		
Intracellular sodium	8/15	mM	$\frac{d}{dt}{\text{Na}}_{\text{x}}^{+}$	$={\text{J}}_{\text{leak},\text{Na}}^{\text{x}}-3{\text{J}}_{\text{pump}}^{\text{x}}+{\text{J}}_{\text{stim}}^{\text{x}}\left(t\right)$	* (A.1)
Neuronal glucose	1.2	mM	$\frac{d}{dt}{\text{GLC}}_{\text{n}}$	$={\text{J}}_{\text{GLC}}^{\text{en}}-{\text{J}}_{\text{HKPFK}}^{\text{n}}$	(A.2)
Astrocytic glucose	1.19	mM	$\frac{d}{dt}{\text{GLC}}_{\text{g}}$	$={\text{J}}_{\text{GLC}}^{\text{cg}}+{\text{J}}_{\text{GLC}}^{\text{eg}}-{\text{J}}_{\text{HKPFK}}^{\text{g}}$	(A.3)
Glyceraldehyde-3-phosphate	0.0046	mM	$\frac{d}{dt}{\text{GAP}}_{\text{x}}$	$=2{\text{ J}}_{\text{HKPFK}}^{\text{x}}-{\text{J}}_{\text{PGK}}^{\text{x}}$	(A.4)
Phosphoenolpyruvate	0.015	mM	$\frac{d}{dt}{\text{PEP}}_{\text{x}}$	$={\text{J}}_{\text{PGK}}^{\text{x}}-{\text{J}}_{\text{PK}}^{\text{x}}$	(A.5)
Pyruvate	0.17	mM	$\frac{d}{dt}{\text{PYR}}_{\text{x}}$	$={\text{J}}_{\text{PK}}^{\text{x}}-{\text{J}}_{\text{LDH}}^{\text{x}}-{\text{J}}_{\text{mito},\text{ in}}^{\text{x}}$	(A.6)
Neuronal lactate	0.6	mM	$\frac{d}{dt}{\text{LAC}}_{\text{n}}$	$={\text{J}}_{\text{LDH}}^{\text{n}}-{\text{J}}_{\text{LAC}}^{\text{ne}}$	(A.7)
Astrocytic lactate	0.6	mM	$\frac{d}{dt}{\text{LAC}}_{\text{g}}$	$={\text{J}}_{\text{LDH}}^{\text{g}}-{\text{J}}_{\text{LAC}}^{\text{ge}}-{\text{J}}_{\text{LAC}}^{\text{gc}}$	(A.8)
Cytosolic NADH ^†^	0.006/0.1	mM	$\frac{d}{dt}{\text{NADH}}_{\text{x}}^{\text{cyto}}$	$={\left(1-\xi \right)}^{-1}\left({\text{J}}_{\text{PGK}}^{\text{x}}-{\text{J}}_{\text{LDH}}^{\text{x}}-{\text{J}}_{\text{shuttle}}^{\text{x}}\right)$	(A.9)
Mitochondrial NADH ^†^	0.12	mM	$\frac{d}{dt}{\text{NADH}}_{\text{x}}^{\text{mito}}$	$=\xi^{-1}\left(4{\text{J}}_{\text{mito},\text{in}}^{\text{x}}-{\text{J}}_{\text{mito},\text{out}}^{\text{x}}+{\text{J}}_{\text{shuttle}}^{\text{x}}\right)$	(A.10)
Neuronal ATP ^‡^	2.2	mM	$\frac{d}{dt}{\text{ATP}}_{\text{n}}^{}$	$=\left(-2{\text{J}}_{\text{HKPFK}}^{\text{n}}+{\text{J}}_{\text{PGK}}^{\text{n}}+{\text{J}}_{\text{PK}}^{\text{n}}-{\text{J}}_{\text{ATPases}}^{\text{n}}-{\text{J}}_{\text{pump}}^{\text{n}}+3.6{\text{J}}_{\text{mito},\text{out}}^{\text{n}}+{\text{J}}_{\text{CK}}^{\text{n}}\right){\left(1-\frac{d{\text{AMP}}_{\text{n}}}{d{\text{ATP}}_{\text{n}}}\right)}^{-1}$	(A.11)
Astrocytic ATP ^†^	2.2	mM	$\frac{d}{dt}{\text{ATP}}_{\text{g}}^{}$	$=\left(-2{\text{J}}_{\text{HKPFK}}^{\text{g}}+{\text{J}}_{\text{PGK}}^{\text{g}}+{\text{J}}_{\text{PK}}^{\text{g}}-{\text{J}}_{\text{ATPases}}^{\text{g}}-\frac{7}{4}{\text{ J}}_{\text{pump}}^{\text{g}}+\frac{3}{4}{\text{J}}_{\text{pump},0}^{\text{g}}+3.6{\text{J}}_{\text{mito},\text{out}}^{\text{g}}+{\text{J}}_{\text{CK}}^{\text{g}}\right){\left(1-\frac{d{\text{AMP}}_{\text{g}}}{d{\text{ATP}}_{\text{g}}}\right)}^{-1}$	(A.12)
Phosphocreatine	4.9	mM	$\frac{d}{dt}{\text{PCr}}_{\text{x}}^{}$	$=-{\text{J}}_{\text{CK}}^{\text{x}}$	(A.13)
Neuronal oxygen	0.028	mM	$\frac{d}{dt}{\text{O}}_{2\text{n}}$	$={\text{J}}_{{\text{O}}_{2}\text{m}}^{\text{cn}}-0.6{\text{J}}_{\text{mito},\text{out}}^{\text{n}}$	(A.14)
Astrocytic oxygen	0.028	mM	$\frac{d}{dt}{\text{O}}_{2\text{g}}$	$={\text{J}}_{{\text{O}}_{2}\text{m}}^{\text{cg}}-0.6{\text{J}}_{\text{mito},\text{out}}^{\text{g}}$	(A.15)
Capillary oxygen	7	mM	$\frac{d}{dt}{\text{O}}_{2\text{c}}$	$={\text{J}}_{{\text{O}}_{2}}^{\text{c}}-1/r_{\text{cn}}{\text{J}}_{{\text{O}}_{2}\text{m}}^{\text{cn}}-1/r_{\text{cg}}{\text{J}}_{{\text{O}}_{2}\text{m}}^{\text{cg}}$	(A.16)
Capillary glucose	4.5	mM	$\frac{d}{dt}{\text{GLC}}_{\text{c}}$	$={\text{J}}_{\text{GLC}}^{\text{c}}-1/r_{\text{ce}}{\text{J}}_{\text{GLC}}^{\text{ce}}-1/r_{\text{cg}}{\text{J}}_{\text{GLC}}^{\text{cg}}$	(A.17)
Capillary lactate	0.55	mM	$\frac{d}{dt}{\text{LAC}}_{\text{c}}$	$={\text{J}}_{\text{LAC}}^{\text{c}}+1/r_{\text{ce}}{\text{J}}_{\text{LAC}}^{\text{ec}}+1/r_{\text{cg}}{\text{J}}_{\text{LAC}}^{\text{gc}}$	(A.18)
Venous volume	0.02		$\frac{d}{dt}{\text{V}}_{\text{v}}$	= *F*_in_ *(t)-F*_out_	*(A.19)
Deoxyhemoglobin	0.058	mM	$\frac{d}{dt}{\text{dHb}}_{}$	$=F_{\text{in}}\left(t\right)\left(O_{2a}-O_{2\bar{c}}\right)-F_{\text{out}}\frac{\text{dHb}}{{\text{V}}_{\text{v}}}$	*(A.20)
Extracellular glucose	2.48	mM	$\frac{d}{dt}{\text{GLC}}_{\text{e}}$	$={\text{J}}_{\text{GLC}}^{\text{ce}}-1/r_{\text{eg}}{\text{J}}_{\text{GLC}}^{\text{eg}}-1/r_{\text{en}}{\text{J}}_{\text{GLC}}^{\text{en}}$	(A.21)
Extracellular lactate	0.6	mM	$\frac{d}{dt}{\text{LAC}}_{\text{e}}$	$=1/r_{\text{en}}{\text{J}}_{\text{LAC}}^{\text{ne}}+1/r_{\text{eg}}{\text{J}}_{\text{LAC}}^{\text{ge}}-{\text{J}}_{\text{LAC}}^{\text{ec}}$	(A.22)
Neuronal membrane voltage	-73	mV	$\frac{d}{dt}\psi_{\text{n}}$	$=C_{m}^{-1}\left(-I_{L}-I_{\text{Na}}-I_{\text{K}}-I_{\text{Ca}}-I_{\text{mAHP}}-I_{\text{pump}}+I_{\text{syn}}\left(t\right)\right)$	*(A.23)
*h* gating variable	0.99		$\frac{d}{dt}h$	$=\frac{\phi_{h}}{\tau_{h}}\left(h_{\infty }-h\right)$	(A.24)
*n* gating variable	0.02		$\frac{d}{dt}n$	$=\frac{\phi_{n}}{\tau_{n}}\left(n_{\infty }-n\right)$	(A.25)
Neuronal calcium	5 10^-5^	mM	$\frac{d}{dt}{\text{Ca}}^{2+}$	$=-\frac{{\text{S}}_{\text{m}}{\text{V}}_{\text{n}}}{F}I_{\text{Ca}}-1/\tau_{\text{Ca}}\left({\text{Ca}}^{2+}-{\text{Ca}}_{0}^{2+}\right)$	(A.26)

*When two values are indicated, the first one corresponds to the neuronal compartment and the second one to the astrocytic compartment.

^†^ NADH stands for nicotinamide adenine dinucleotide.

^‡^ ATP stands for adenosine triphosphate.

All the fluxes and currents appearing in Table 1, as well the equations describing the dynamics of the gating variables, are given in Table 2. Like for Table 1, these equations are taken from [40, 44–46] except equations (A.35)-(A.37) which are original. All rates and state variables are given per unit cell volume (neuron or astrocyte) or per unit capillary volume to the exception of J_GLC_^ce^, J_LAC_^ec^, LAC_e_ and GLC_e_ that are given per unit extracellular volume. Mitochondrial and cytosolic NADH levels are given per unit mitochondrial or cytosolic volume respectively.

**Table 2:** Rates, transports and currents.

**Reaction, transport or current**	**Equation**		
Sodium leak	${\text{J}}_{\text{leak},\text{Na}}^{\text{x}}$	$=\frac{{\text{S}}_{\text{m}}{\text{V}}_{\text{x}}}{F}g_{\text{Na}}^{\text{x}}\left[\frac{RT}{F}\log\left({\text{Na}}_{\text{e}}^{+}/{\text{Na}}_{\text{x}}^{+}\right)-\psi_{\text{x}}\right]$	(A.27)
Na, K-ATPase	${\text{J}}_{\text{pump}}^{\text{x}}$	$={\text{S}}_{\text{m}}{\text{V}}_{\text{x}}k_{\text{pump}}^{\text{x}}{\text{ ATP}}_{\text{x}}{\text{Na}}_{\text{x}}^{+}{\left(1+\frac{{\text{ ATP}}_{\text{x}}}{K_{\text{m},\text{pump}}}\right)}^{-1}$	(A.28)
Glucose transport	${\text{J}}_{\text{GLC}}^{\text{xy}}$	$=T_{\text{max},\text{ GLC}}^{\text{xy}}\left(\frac{{\text{GLC}}_{\text{x}}}{{\text{GLC}}_{\text{x}}+K_{\text{t},\text{ GLC}}^{\text{xy}}}-\frac{{\text{GLC}}_{\text{y}}}{{\text{GLC}}_{\text{y}}+K_{\text{t},\text{ GLC}}^{\text{xy}}}\right)$	(A.29)
Hexokinase-phosphofructokinase	${\text{J}}_{\text{HKPFK}}^{\text{x}}$	$=k_{\text{HKPFK}}^{\text{x}}{\text{ ATP}}_{\text{x}}\frac{{\text{GLC}}_{\text{x}}}{{\text{GLC}}_{\text{x}}+K_{\text{g}}^{}}{\left[1+{\left(\frac{{\text{ATP}}_{\text{x}}}{K_{\text{I},\text{ATP}}}\right)}^{nH}\right]}^{-1}$	(A.30)
Phosphoglycerate kinase	${\text{J}}_{\text{PGK}}^{\text{x}}$	$=k_{\text{PGK}}^{\text{x}}{\text{ GAP}}_{\text{x}}{\text{ ADP}}_{\text{x}}\left(N-{\text{NADH}}_{\text{x}}^{\text{cyto}}\right)/{\text{NADH}}_{\text{x}}^{\text{cyto}}$	(A.31)
Pyruvate kinase	${\text{J}}_{\text{PK}}^{\text{x}}$	$=k_{\text{PK}}^{\text{x}}{\text{ PEP}}_{\text{x}}{\text{ ADP}}_{\text{x}}$	(A.32)
Lactate dehydrogenase	${\text{J}}_{\text{LDH}}^{\text{x}}$	$=k_{\text{LDH}}^{\text{x}+}{\text{ PYR}}_{\text{x}}{\text{ NADH}}_{\text{x}}^{\text{cyto}}-k_{\text{LDH}}^{\text{x}-}{\text{ PYR}}_{\text{x}}\left(N-{\text{NADH}}_{\text{x}}^{\text{cyto}}\right)$	(A.33)
Lactate transport	${\text{J}}_{\text{LAC}}^{\text{xy}}$	$=T_{\text{max},\text{ LAC}}^{\text{xy}}\left(\frac{{\text{LAC}}_{\text{x}}}{{\text{LAC}}_{\text{x}}+K_{\text{t},\text{ LAC}}^{\text{xy}}}-\frac{{\text{LAC}}_{\text{y}}}{{\text{LAC}}_{\text{y}}+K_{\text{t},\text{ LAC}}^{\text{xy}}}\right)$	(A.34)
TCA cycle	${\text{J}}_{\text{mito},\text{in}}^{\text{x}}$	$=V_{\text{max},\text{ in}}^{\text{x}}\frac{{\text{PYR}}_{\text{x}}}{{\text{PYR}}_{\text{x}}+K_{\text{m}}^{\text{mito}}}\frac{N-{\text{NADH}}_{\text{x}}^{\text{mito}}}{N-{\text{NADH}}_{\text{x}}^{\text{mito}}+K_{\text{m},\text{NAD}}^{\text{x}}}$	(A.35)
Electron transport chain	${\text{J}}_{\text{mito},\text{out}}^{\text{x}}$	$=V_{\text{max},\text{ out}}^{\text{x}}\frac{{\text{O}}_{2\text{x}}}{{\text{O}}_{2\text{x}}+K_{{\text{O}}_{2}}^{\text{mito}}}\frac{{\text{ADP}}_{\text{x}}}{{\text{ADP}}_{\text{x}}+K_{\text{m},\text{ADP}}^{\text{x}}}\frac{{\text{NADH}}_{\text{x}}^{\text{mito}}}{{\text{NADH}}_{\text{x}}^{\text{mito}}+K_{\text{m},\text{NADH}}^{\text{x}}}$	(A.36)
NADH shuttles	${\text{J}}_{\text{shuttle}}^{\text{x}}$	$=T_{\text{NADH}}^{\text{x}}\frac{R_{\text{x}}^{-}}{R_{\text{x}}^{-}+M_{\text{x}}^{\text{cyto}}}\frac{R_{\text{x}}^{+}}{R_{\text{x}}^{+}+M_{\text{x}}^{\text{mito}}}$	^†^ (A.37)
Creatine kinase	${\text{J}}_{\text{CK}}^{\text{x}}$	$=k_{\text{CK}}^{\text{x}+}{\text{ ADP}}_{\text{x}}^{}{\text{PCr}}_{\text{x}}-k_{\text{CK}}^{\text{x}-}{\text{ ATP}}_{\text{x}}\left(C-{\text{PCr}}_{\text{x}}^{}\right)$	(A.38)
Oxygen exchange	${\text{J}}_{{\text{O}}_{2}\text{m}}^{\text{cx}}$	$=\frac{PS_{\text{cap}}}{V_{\text{x}}}\left({\text{K}}_{{\text{O}}_{2}}{\left(\frac{Hb.OP}{O_{2c}}-1\right)}^{-1/nh}-O_{2n}\right)$	(A.39)
Capillary oxygen flow	${\text{J}}_{{\text{O}}_{2}}^{\text{c}}$	$=\frac{2F_{\text{in}}\left(t\right)}{V_{\text{cap}}}\left({\text{O}}_{2\text{a}}-{\text{O}}_{2\text{c}}\right)$	(A.40)
Capillary glucose flow	${\text{J}}_{\text{GLC}}^{\text{c}}$	$=\frac{2F_{\text{in}}\left(t\right)}{V_{\text{cap}}}\left({\text{GLC}}_{\text{a}}-{\text{GLC}}_{\text{c}}\right)$	(A.41)
Capillary lactate flow	${\text{J}}_{\text{LAC}}^{\text{c}}$	$=\frac{2F_{\text{in}}\left(t\right)}{V_{\text{cap}}}\left({\text{LAC}}_{\text{a}}-{\text{LAC}}_{\text{c}}\right)$	(A.42)
Oxygen concentration at the end of the capillary	${\text{O}}_{2\bar{\text{c}}}$	$=2{\text{O}}_{2\text{c}}-{\text{O}}_{2\bar{\text{c}}}$	(A.43)
Leak current	*I*_L_	$=g_{L}\left(\psi_{\text{n}}-E_{L}\right)$	(A.44)
Sodium current	*I*_Na_	$=g_{\text{Na}}m_{\infty }^{3}h\left(\psi_{\text{n}}-\frac{RT}{F}\log\left({\text{Na}}_{\text{e}}^{+}/{\text{Na}}_{\text{n}}^{+}\right)\right)$	^‡^ (A.45)
Potassium current	*I*_K_	$=g_{\text{K}}n^{4}\left(\psi_{\text{n}}-E_{\text{K}}\right)$	^‡^ (A.46)
Calcium current	*I*_Ca_	$=g_{\text{Ca}}m_{\text{Ca}}^{2}\left(\psi_{\text{n}}-E_{\text{Ca}}\right)$	^‡^ (A.47)
Calcium-dependent potassium current	*I*_mAHP_	$=g_{\text{mAHP}}\frac{{\text{Ca}}^{2+}}{{\text{Ca}}^{2+}+K_{D}}\left(\psi_{\text{n}}-E_{\text{K}}\right)$	(A.48)
Na, K-ATPase current	*I*_pump_	$=Fk_{\text{pump}}^{\text{n}}{\text{ATP}}_{\text{n}}\left({\text{Na}}_{\text{n}}^{+}-{\text{Na}}_{0}^{+}\right){\left(1+\frac{{\text{ATP}}_{\text{x}}}{K_{\text{m},\text{pump}}}\right)}^{-1}$	(A.49)
Flow out of the venous balloon	*F*_out_	$=F_{0}\left[{\left(\frac{{\text{V}}_{\text{v}}}{{\text{V}}_{\text{v}0}}\right)}^{1/\alpha_{\text{v}}}+\frac{\tau_{\text{V}}}{{\text{V}}_{\text{v}0}}{\left(\frac{{\text{V}}_{\text{v}}}{{\text{V}}_{\text{v}0}}\right)}^{-1/2}\frac{d{\text{V}}_{\text{v}}}{dt}\right]$	(A.50)

^†^ With $R_{\text{x}}^{-}={\text{NADH}}_{\text{x}}^{\text{cyto}}/\left(N-{\text{NADH}}_{\text{x}}^{\text{cyto}}\right)$ and $R_{\text{x}}^{+}=\left(N-{\text{NADH}}_{\text{x}}^{\text{mito}}\right)/{\text{NADH}}_{\text{x}}^{\text{mito}}$.

^‡^ Further equations in the Hodgkin-Huxley model are: $\alpha_{m}=-0.1\left(\psi_{\text{n}}+33\right)/\left(\exp\left[-0.1\left\{\psi_{\text{n}}+33\right\}\right]-1\right)$, $\beta_{m}=4\exp\left[-\left\{\psi_{\text{n}}+58\right\}/12\right]$, $\alpha_{h}=0.07\exp\left[-\left\{\psi_{\text{n}}+50\right\}/10\right]$, $\beta_{h}=1/\left(\exp\left[-0.1\left\{\psi_{n}+20\right\}\right]+1\right)$, $\alpha_{n}=-0.01\left(\psi_{\text{n}}+34\right)/\left(\exp\left[-0.1\left\{\psi_{\text{n}}+34\right\}\right]-1\right)$, $\beta_{n}=0.125\exp\left[-\left\{\psi_{\text{n}}+44\right\}/25\right]$, $m_{\infty }=\alpha_{m}{\left(\alpha_{m}+\beta_{m}\right)}^{-1}$, $n_{\infty }=\alpha_{n}{\left(\alpha_{n}+\beta_{n}\right)}^{-1}$, $h_{\infty }=\alpha_{h}{\left(\alpha_{h}+\beta_{h}\right)}^{-1}$, $\tau_{n}=10^{-3}{\left(\alpha_{n}+\beta_{n}\right)}^{-1}$, $\tau_{h}=10^{-3}{\left(\alpha_{h}+\beta_{h}\right)}^{-1}$, $m_{\text{Ca}}=1/\left(1+\exp\left[-\left\{\psi_{\text{n}}+20\right\}/9\right]\right)$ and $E_{L}={\left(g_{\text{K}}^{\text{pas}}+g_{\text{Na}}^{\text{n}}\right)}^{-1}\left[g_{\text{K}}^{\text{pas}}E_{K}+g_{\text{Na}}^{\text{n}}\frac{RT}{F}\log\left({\text{Na}}_{\text{e}}^{+}/{\text{Na}}_{\text{n}}^{+}\right)\right]$.

### Additional equations

ADP_x_ is given as a function of the ATP concentration (*x* stands for *n* or *g*). It reads:

$$ADP_{x}=\frac{ATP_{x}}{2}[-q_{AK}+\sqrt{q_{AK}^{2}+4q_{AK}(A/ATP_{x}-1)}]$$ (1)

with A = AMP_x_+ADP_x_+ATP_x_ = 2.212 mM the total adenine nucleotide concentration and *q_AK_* = 0.92 the adenylate kinase equilibrium constant [40, 45]. As a consequence:

$$\frac{dAMP_{x}}{dATP_{x}}=-1+\frac{q_{AK}}{2}-\frac{1}{2}\sqrt{u_{x}}+\frac{q_{AK*A}}{ATP_{x}\sqrt{u_{x}}}$$ (2)

with u_x_ = q^2^_AK_+4·q_AK_·(A/ATP_x_-1).

### Input to the model

The model receives input from a presynaptic excitatory population. Glutamate released by excitatory presynaptic neurons drives the intracellular sodium concentration in neurons and astrocytes and activates AMPA receptors on neurons, thus inducing a synaptic current *I_syn_*. The presynaptic population contains *N_exc_* excitatory neurons discharging at frequency *f*_exc_(t). This presynaptic population thus generates an excitatory conductance *g*_exc_(t) given by:

$$g_{exc}(t)=N_{exc}\bar{g}f_{exc}(t)$$ (3)

with g = 7.8·10^-6^ mS·cm^-2^·sec the total surface under the conductance evoked by one excitatory event [47, 48]. The corresponding synaptic current is then given by:

$$I_{syn}(t)=g_{exc}(t)(\psi_{n}-E_{AMPA})$$ (4)

with *ψ_n_* the neuronal membrane voltage and *E*_AMPA_ = 0 mV the reversal potential of AMPA ionotropic receptors. It is estimated that about two thirds of the current generated at AMPA receptors is due to a flow of sodium ions [49]. Sodium also flows through voltage-dependent sodium channels when the neuron is active (I_Na_). As a consequence, the sodium drive to the neuron is approximated by:

$$J_{stim}^{n}=\frac{S_{m}V_{n}}{F}(\frac{2}{3}I_{syn}-I_{Na})$$ (5)

with S_m_·V_n_ = 2.5·10^4^ cm^-1^ the ratio between neuronal membrane surface and neuronal volume and F = 9.64853·10^4^ C·mol^-1^ the Faraday constant. Finally, the glutamate is cleared from the synaptic cleft by excitatory amino acid transporters located on the astrocyte membrane. Those transporters use the electrochemical sodium gradient to transport glutamate with a stoichiometry of three sodium ions for one glutamate molecule. We thus write the sodium drive to the astrocyte as follows:

$$J_{stim}^{g}=3\Delta_{glut}N_{exc}f_{exc}(t)$$ (6)

with ∆_glut_ = 2.25·10^-5^ mM a constant, which corresponds to the total amount of glutamate released in the synaptic cleft by each presynaptic action potential multiplied by the ratio between synaptic and astrocytic fractional volumes. For the sake of simplicity, we assume that *f*_exc_(t) always follows the same temporal dynamics exponentially decaying from *f*_0_ = 3.2 Hz to *f*_∞_ = 0.5 Hz with a time constant t_*f*_ = 2.5 sec and *N*_exc_ = 1500.

### Cerebral blood flow

Following *in vivo* measurements in rodents [50, 51], the cerebral blood flow is modeled as a piecewise double exponential function delayed in time by *t*_1_ relatively to the onset of stimulation *t*_0_. It reads:

F(t)={F0{F01.1+1.5[exp(−t−t15)−exp(t−t12)]F0+[F(tend)−F0]exp(−t−tend5)}ift<t1t1≤t≤tendt<tend} (7)

with *F*_0_ = 0.012 sec^-1^ [46]. Typical values are *t*_0_ = 0 sec and *t*_0_ = 1 sec, *t*_end_ being the time at which stimulation ends. Two distinct simulation scenarios are considered to mimic *in vitro* and *in vivo* conditions. In the *in vivo* scenario, Equation (7) is used while in the *in vitro* scenario, the capillary state variables remain constant at their steady-state while the rest of the variables are left free to vary. Our simulations have shown that this is almost equivalent to taking a constant blood flow *F*(t) = *F*_0_.

### BOLD signal

The blood-oxygen-level-dependent (BOLD) signal is computed following [52]. It is written as a function of the deoxyhemoglobin concentration (dHb) and of the venous volume (V_v_):

$$BOLD(t)=V_{V,0}[(k_{1}+k_{2})(1-\frac{dHb}{dHb_{0}})-(k_{2}+k_{3})(1-\frac{V_{V}}{V_{V,0}})]$$ (8)

with dimensionless parameters *k*_1_ = 2.22, *k*_2_ = 0.46 and *k*_3_ = 0.43 [40]. The steady-state values of deoxyhemoglobin (dHb_0_) and venous volume (V_v,0_) are given in Table A1.

### Optimization procedure

As noted already by Aubert and colleagues [53], most models of energy metabolism concentrate on erythrocytes, muscles or other organs such as the liver. It is also not clear whether or not parameters drawn from experiments could be directly injected as such into a model without spatial dimensions and without diffusion processes like ours. To circumvent this problem, we proceeded as follows:

First, we chose target steady-state values for the concentration of metabolites following measures reported in the literature. Specifically, we chose the concentration of intracellular sodium following [54], the concentration of intracellular glucose, phosphoenolpyruvate, pyruvate, adenosine triphosphate and phosphocreatine following [55], and glyceraldehyde-3-phosphate following [56]. Finally, the NADH concentration in all four compartments where it appears in the model was chosen following [56] and calculations based on results by Kasischke and colleagues [23].

We then optimized a subset of model parameters (see Table 3) by fitting its predictions to the temporal dynamics of NADH fluorescence as measured by Kasischke et al. [23]. Namely, the dynamics of the NADH concentration in various compartments was extracted empirically from Fig. 4D in [23]. Data points were then fitted with sums of exponentials in order to obtain continuous curves. We then optimized the model by minimizing the distance between the temporal dynamics of NADH in the model and the one in the smoothed curve obtained from [23] using least-square distance as the error measure and using the downhill simplex algorithm. After the optimization converged, we rounded the value of the optimal parameter set and recomputed the steady-state value. All along optimization, we checked that the steady-state was stable by computing its Jacobian matrix (first order approximation) [45]. The parameter set in Table 3 is the set resulting from this procedure.

**Table 3:** Parameters.

**Fixed parameters**	
Volume fractions	V_e_ = 0.2, V_cap_ = 0.0055, V_g_ = 0.25, V_n_ = 0.45, ξ = 0.07, *r*_en_ = V_e_/V_n_, *r*_eg_ = V_e_/V_g_, *r*_ce_ = V_cap_/V_e_, *r*_cg_ = V_cap_/V_g_, *r*_cn_ = V_cap_/V_n_
Surface-to-volume ratios	S_m_V_n_ = 2.5 10^4^, S_m_V_g_ = 2.5 10^4^ cm^-1^
Physical constants	*R* = 8.31451 J mol^-1^ K^-1^, *F* = 9.64853 10^4^ C mol^-1^, *RT*/*F* = 26.73 mV, $\psi_{\text{g}}=-70$mV, ${\text{Na}}_{\text{e}}^{+}=150$mM
Glucose exchange affinities	$K_{\text{t},\text{GLC}}^{\text{en}}=8$, $K_{\text{t},\text{GLC}}^{\text{eg}}=8$, $K_{\text{t},\text{GLC}}^{\text{cg}}=8$, $K_{\text{t},\text{GLC}}^{\text{ce}}=8$mM
Lactate exchange affinities	$K_{\text{t},\text{LAC}}^{\text{en}}=0.74$, $K_{\text{t},\text{LAC}}^{\text{ge}}=3.5$, $K_{\text{t},\text{LAC}}^{\text{gc}}=1$, $K_{\text{t},\text{LAC}}^{\text{ec}}=1$mM
Hexokinase-phosphofructokinase system	*K*_I, ATP_ = 1 mM, *nH* = 4, *K*_g_ = 0.05 mM
Oxygen exchange constants	$K_{{\text{O}}_{2}}^{}=0.0361$mM, *Hb.OP* = 8.6 mM, *nh* = 2.73
Electron transport chain	$K_{{\text{O}}_{2}}^{\text{mito}}=0.001$mM
Hodgkin-Huxley parameters	*C_m_* = 10^-3^ mF cm^-2^, *g_L_* = 0.02, *g*_Na_ = 40, *g*_K_ = 18, *g*_Ca_ = 0.02, *g*_mAHP_ = 6.5 mS cm^-2^, *K_D_* = 30 10^-3^ mM, $\tau_{\text{Ca}}=150\;10^{-3}$s, ${\text{Ca}}_{0}^{2+}=0.5\;10^{-4}$mM, *E*_K_ = −80, *E*_Ca_ = 120 mV, $\phi_{n}=\phi_{h}=4$
Venous balloon	$\tau_{\text{v}}=35$s, $\alpha_{\text{v}}=0.5$
Blood flow contribution to capillary glucose and oxygen	O_2a_ = 8.35, GLC_a_ = 4.75 mM
Na, K-ATPase and sodium leak	$g_{\text{Na}}^{\text{n}}=0.0136$, $g_{\text{Na}}^{\text{g}}=0.0061$, $g_{\text{K}}^{\text{pas}}=0.2035$mS cm^-2^, $k_{\text{pump}}^{\text{n}}=2.210^{-6}$, $k_{\text{pump}}^{\text{g}}=4.510^{-7}$cm mM^-1^ s^-1^, ${\text{J}}_{\text{pump},0}^{\text{g}}$= 0.0687 mM s^-1^, *K*_m, pump_ = 0.5 mM
Total creatine plus phosphocreatine concentration	*C* = 10 mM
Total nicotinamide adenine dinucleotide concentration	*N* = 0.212 mM
TCA cycle	$K_{\text{m}}^{\text{mito}}=0.04$mM
**Optimized parameters**	
Lactate dehydrogenase	$k_{\text{LDH}}^{\text{n}+}=72.3$, $k_{\text{LDH}}^{\text{g}+}=1.59$mM^-1^ s^-1^
NADH shuttles	$M_{\text{n}}^{\text{cyto}}=4.910^{-8}$, $M_{\text{g}}^{\text{cyto}}=2.510^{-4}$, $M_{\text{n}}^{\text{mito}}=3.9310^{5}$, $M_{\text{g}}^{\text{mito}}=1.0610^{4}$
Electron transport chain	$K_{\text{m},\text{ADP}}^{\text{n}}=3.4110^{-3}$, $K_{\text{m},\text{ADP}}^{\text{g}}=0.48310^{-3}$, $K_{\text{m},\text{NADH}}^{\text{n}}=4.4410^{-2}$, $K_{\text{m},\text{NADH}}^{\text{g}}=2.6910^{-2}$mM
Creatine kinase	$k_{\text{CK}}^{\text{n}+}=0.0433$, $k_{\text{CK}}^{\text{g}+}=0.00135$mM^-1^ s^-1^
TCA cycle	$K_{\text{m},\text{NAD}}^{\text{n}}=0.409$, $K_{\text{m},\text{NAD}}^{\text{g}}=40.3$mM
**Constrained parameters**	
Glucose exchange constants	$T_{\text{max},\text{GLC}}^{\text{en}}=0.041$, $T_{\text{max},\text{GLC}}^{\text{ce}}=0.239$, $T_{\text{max},\text{GLC}}^{\text{eg}}=0.147$, $T_{\text{max},\text{GLC}}^{\text{cg}}=0.0016$mM s^-1^
Lactate exchange constants	$T_{\text{max},\text{LAC}}^{\text{gc}}=0.00243$, $T_{\text{max},\text{LAC}}^{\text{ne}}=24.3$, $T_{\text{max},\text{LAC}}^{\text{ge}}=106.1$, $T_{\text{max},\text{LAC}}^{\text{ec}}=0.25$mM s^-1^
Hexokinase-phosphofructokinase system	$k_{\text{HKPFK}}^{\text{n}}=0.0504$, $k_{\text{HKPFK}}^{\text{g}}=0.185$s^-1^
Lactate dehydrogenase	$k_{\text{LDH}}^{\text{n}-}=0.72$, $k_{\text{LDH}}^{\text{g}-}=0.071$mM^-1^ s^-1^
Oxygen exchange constants	$\frac{PS_{\text{cap}}}{V_{\text{n}}}=1.66$, $\frac{PS_{\text{cap}}}{V_{\text{g}}}=0.87$s^-1^
Electron transport chain	$V_{\text{max},\text{out}}^{\text{n}}=0.164$, $V_{\text{max},\text{out}}^{\text{g}}=0.064$mM s^-1^
TCA cycle	$V_{\text{max},\text{in}}^{\text{n}}=0.1303$, $V_{\text{max},\text{in}}^{\text{g}}=5.7$mM s^-1^
Phosphoglycerate kinase	$k_{\text{PGK}}^{\text{n}}=3.97$, $k_{\text{PGK}}^{\text{g}}=135.2$mM^-1^ s^-1^
Pyruvate kinase	$k_{\text{PK}}^{\text{n}}=36.7$, $k_{\text{PK}}^{\text{g}}=401.7$mM^-1^ s^-1^
ATPases	${\text{J}}_{\text{ATPases}}^{\text{n}}=0.1695$, ${\text{J}}_{\text{ATPases}}^{\text{g}}=0.1404$mM s^-1^
Creatine kinase	$k_{\text{CK}}^{\text{n}-}=0.00028$, $k_{\text{CK}}^{\text{g}-}=10^{-5}$mM^-1^ s^-1^
NADH shuttles	$T_{\text{NADH}}^{\text{n}}=10330$, $T_{\text{NADH}}^{\text{g}}=150$mM s^-1^
Blood flow contribution to capillary lactate	LAC_a_ = 0.506 mM

### Numerics

The simulations were run in MATLAB (The Mathworks, Natick MA, USA). The model was integrated with the ordinary differential equation solver with fixed and optimized parameters (ode15s) that is adapted to stiff systems. We used a time step ∂t = 10^-4^ sec when the neuron is spiking and ∂t = 1 sec starting one second after the end of presynaptic stimulation. ∂t = 10^-4^ sec is smaller than the fastest time constant appearing in the Hodgkin-Huxley equations [tau_h_(-80 mV) = 6.4·10^-4^ sec]. The second time step (∂t = 1 sec) is small enough for the slow metabolic processes and maintains simulation time and memory usage to reasonable values for an average desktop PC. Simulations take a couple of minutes to execute on a recent laptop.

### Results

We developed a model of the coupling between neuronal activity and metabolic response in neurons and astrocytes. The model employed to simulate the neural-glial-vascular (NGV) functional system is composed of four distinct computational units representing a neuron, an astrocyte, a capillary and the extracellular space (Fig. 1). The core of our model is composed of the compartmentalized model of brain energy metabolism recently proposed by Aubert and Costalat [13, 40]. This model connects a model of erythrocyte glycolytic metabolism [45, 56] together with the so-called “Balloon model” of blood flow dynamics [46]. From this starting point, we added a precise description of neuronal membrane excitability formulated within the Hodgkin-Huxley framework [57]. Channels dynamics is drawn from a model proposed by Wang [44]. It includes all the standard Hodgkin-Huxley currents plus a high-threshold calcium current and a calcium-gated potassium current inducing spike-frequency adaptation. The Hodgkin-Huxley model is connected to the metabolic pathways through the electrogenic Na, K-ATPase pump which is responsible for a net outward current and concomitant ATP consumption. We modified the metabolic pathways to include compartmentalization of NADH between the cytosol and mitochondria. To do so, we developed a very simple model of mitochondrial respiration and added NADH malate-aspartate shuttles between the cytosol and mitochondria, drawing inspiration from a model by [58]. Finally, the model is driven by external input modeled as a global excitatory presynaptic activity and coordinated increase of the cerebral blood flow. The presynaptic population is coarsely described through a time-dependent excitatory conductance. This conductance drives sodium flow in neurons through AMPA receptors and action potential-generating voltage-gated sodium channels, and in astrocytes through excitatory amino acid transporters which co-transport glutamate using the sodium gradient. The model is illustrated in Fig. 1 and extensively described in the Methods section.

**Figure 2:**
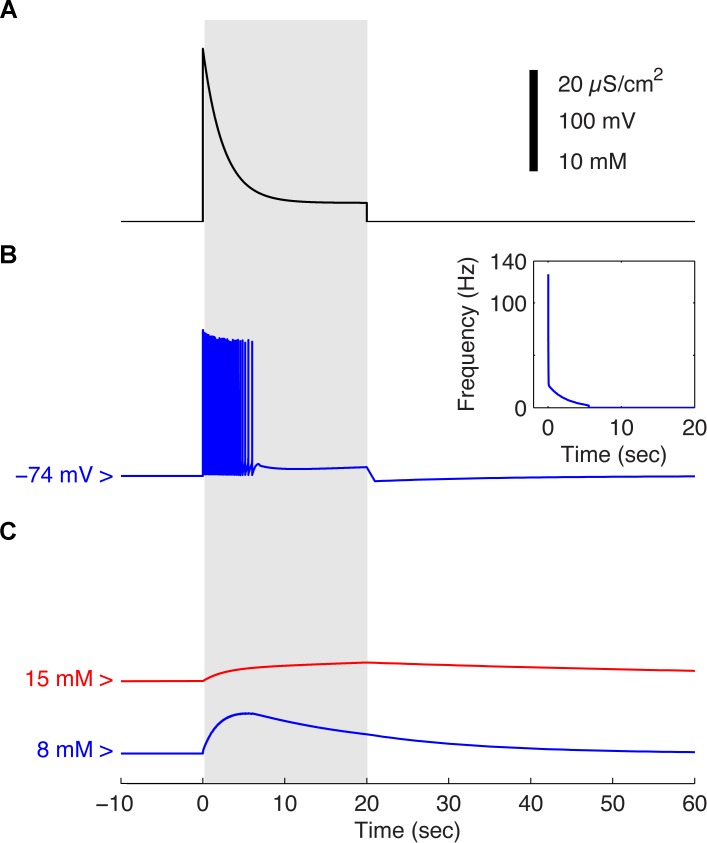
Simulated dynamics of Hodgkin-Huxley equations and of intracellular sodium concentrations during an *in vitro* 20 sec stimulation episode. **A**. Time course of the global excitatory conductance simulating the activation of AMPA receptors by the presynaptic population. The gray area denotes the stimulation period. **B**. Neuronal membrane voltage recorded in response to the excitatory stimulation plotted in A and instantaneous firing rate (inset). **C**. Sodium concentration in the neuronal cytosol (blue) and in the astrocytic cytosol (red) recorded in response to the excitatory stimulation plotted in A

We first tested the model for its responsiveness to an excitatory stimulus (Fig. 2A) and recorded its voltage response (Fig. 2B). In response to this stimulus, the model generated action potentials within the initial 7 sec of the stimulation. Because of spike-frequency adaptation and of the time course of the excitatory stimulus, the frequency of elicited action potentials quickly decreased until the neuron eventually ceased to fire (Fig. 2B inset). Response trajectories of intracellular sodium, in both the astrocyte (red) and neuron (blue), showed significant differences in both amplitude and duration, with astrocytes exhibiting a smaller but more sustained response and a delayed recovery (Fig. 2C).

**Figure 3:**
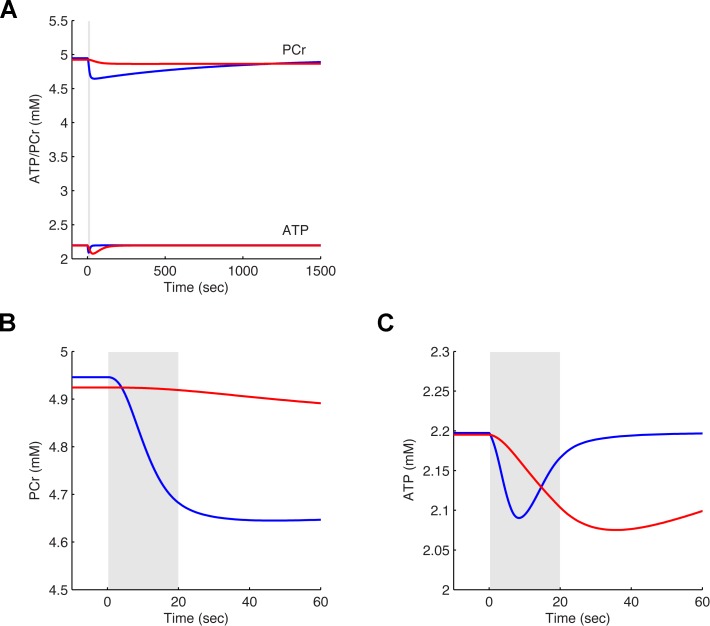
Simulated dynamics of ATP and PCr concentrations during an *in vitro* 20 sec stimulation episode. **A**. Concentration of phosphocreatine (PCr; upper lines at ~5.0 mM) and adenosine triphosphate (ATP; lower lines at ~2.2 mM) in the neuronal (blue) and astrocytic compartments (red) during a 20 sec *in vitro* stimulation episode (same simulation as in Fig. 2). **B**. Zoom-in re-scaling of the area of interest in panel A for PCr in neuronal (blue) and astrocytic compartments (red). **C**. Zoom-in re-scaling of the area of interest in panel A for ATP in neuronal (blue) and astrocytic compartments (red). In panels A, B and C, the gray area denotes the stimulation period

We then examined the time course of critical intermediates in energy metabolism in response to the same excitatory stimulus. We first focused on the concentration changes of adenosine triphosphate (ATP) and phosphocreatine (PCr) in the glial and neuronal compartments (Fig. 3). In both cases, the response to the excitatory stimulation, evidenced as a consumption of these energy rich metabolites, was slower in the astrocytic (red) than in the neuronal compartment (blue) (Fig. 3A). And while the decrease in glial ATP surpassed that seen in the neuron (Fig. 3C), the consumption of PCr predominated in the neuron (Fig. 3B). In both compartments, the resulting decrease in ATP concentration was very limited.

### NADH responses evoked *in vitro* are consistent with the ANLS hypothesis

**Figure 4:**
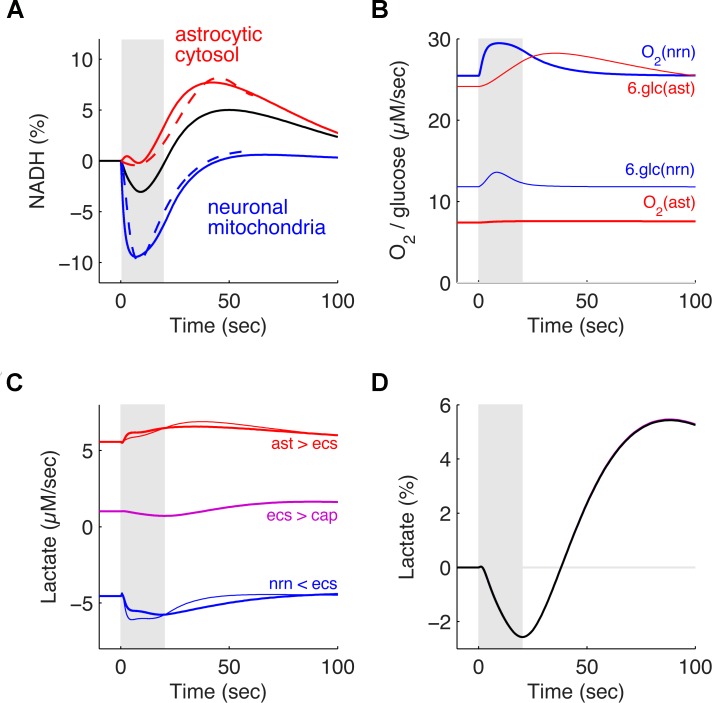
Simulated dynamics of NADH concentrations, of lactate concentrations and of glucose and oxygen consumption during an *in vitro* 20 sec stimulation episode. **A**. Relative fluctuations of the NADH concentration in the astrocytic cytosol (red), in the neuronal mitochondria (blue) and averaged over the whole tissue (black) as evoked by a 20 sec stimulation episode *in vitro* (grey area; same simulation as in Fig. 2 and 3). The dotted lines indicate corresponding *in vitro* data reproduced from Kasischke et al. [23]. **B**. Oxygen utilization (thick lines) and glucose utilization (thin lines) by neurons (blue) and astrocytes (red) as evoked by the same 20 sec stimulation episode as in A. Glucose utilization is multiplied by 6 to allow a direct comparison to oxygen utilization. **C**. Net transport of lactate between the four compartments of the model during the same 20 sec stimulation episode as in A. Lactate is exported by the astrocyte to the extracellular space (thick red line) and imported by the neuron from the extracellular space (thick blue line; net import is negative by convention in this model). The thin red line denotes the activity of the lactate dehydrogenase converting pyruvate into lactate in the astrocytic cytosol while the thin blue line denotes the activity of the lactate dehydrogenase converting lactate into pyruvate in the neuronal cytosol (again, the negative sign is a convention). Note how increase in lactate-to-pyruvate conversion precedes the increase in net lactate import by neurons, while pyruvate-to-lactate conversion follows the increase in net lactate export by astrocytes. **D**. Relative fluctuations of tissue lactate (pink) and of extracellular lactate (black) during the same 20 sec stimulation episode as in A to C. Both lines are superimposed and barely distinguishable

Next, we examined the trajectories of nicotinamide adenine dinucleotide (NADH) in response to the stimulation in the astrocytic, neuronal and mitochondrial compartments (Fig. 4A). The dashed lines represent experimental data from Kasischke et al. [23]. Fig. 4A shows the temporal evolution of the concentration of NADH in the astrocytic cytosol, in the neuronal mitochondria and averaged over the whole tissue as evoked by a 20 sec stimulation episode (see Methods). All three curves are in excellent quantitative agreement with the results reported by Kasischke et al. [23]. In particular, the NADH concentration in the neuronal mitochondria displays an initial dip of about-10% indicating a strong increase of the oxidative metabolism in neurons (Fig. 4B). It then returns towards its baseline before the presynaptic bombardment has finished and finally slightly overshoots in the poststimulus period. On the contrary, the NADH concentration in the astrocytic cytosol increases significantly only about 10 sec after the onset of the stimulation and displays a long-lasting monophasic behavior. This corresponds to a strong and sustained increase of the glycolysis in this compartment (Fig. 4B).

The initial dip in the neuronal mitochondria is the result of consumption of NADH to produce ATP. A recovery and rebound results when NADH is produced from the consumption of lactate imported into the neuronal cytosol from the extracellular space (Fig. 4C). In both Fig. 4B and C, it can be seen that the astrocytic response is slower than the neuronal response. In particular, both oxygen and glucose consumption increase immediately at the beginning of the stimulation in the neuronal compartment. Partially supporting this metabolic activity, neurons immediately start to import lactate from the extracellular space (Fig. 4C). On the contrary, the increase in glucose consumption by astrocytes (Fig. 4B) is more gradual and the increase in lactate export by astrocytes to the extracellular space is slightly delayed (Fig. 4C). The initial release of presynaptic glutamate with subsequent neuronal activity and reuptake into astrocytes lead to the increase in intracellular sodium concentration and activation of the Na, K-ATPase imposing, along with the conversion of glutamate to glutamine in astrocytes, an increased metabolic demand. However, as can be seen in Fig. 2C, the increase in intracellular sodium is slower and more gradual in the astrocytic compartment leading to the 10 second delay in the glial cell metabolic response to the stimulation. Finally, the dynamics of tissue NADH (Fig. 4A) is mirrored in the predicted tissue and extracellular lactate concentrations (Fig. 4D).

The utilization of glucose and oxygen by neurons and astrocytes during this 20 sec stimulation episode is shown in Fig. 4B. For model optimization, we imposed that the largest fraction of glucose goes to astrocytes while the largest fraction of oxygen goes to neurons [42]. We observed that this bias is further increased during stimulation. The neuronal oxygen utilization immediately increased at the onset of stimulation in register with the initial dip of the NADH in the neuronal mitochondria. This is consistent with reports that the astrocytic fraction of glucose utilization increases during stimulation [19].

### The biophysical model correctly predicts rodent *in vivo* oxygen and lactate evoked responses

**Figure 5:**
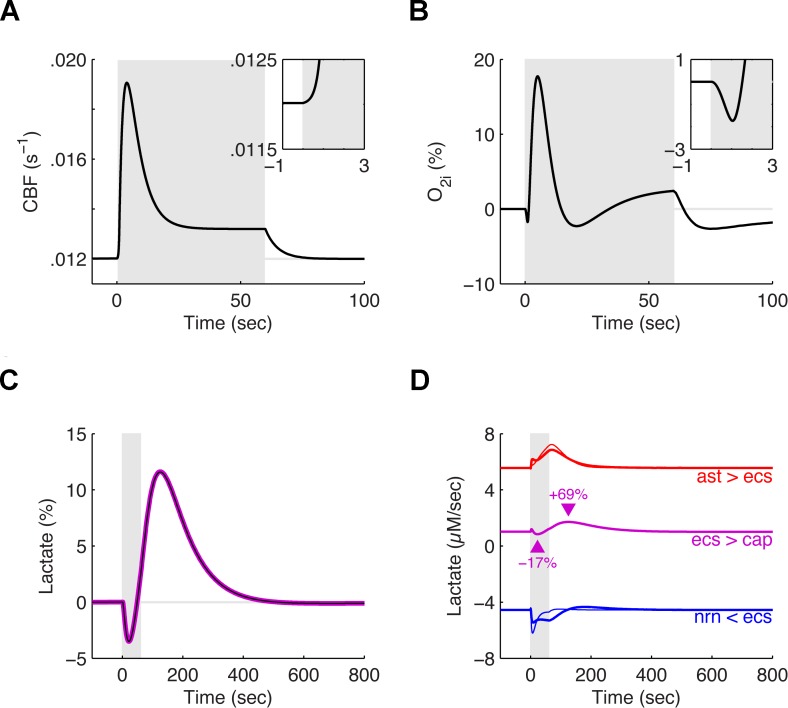
Predicted evoked responses of tissue lactate and of tissue oxygen *in vivo* in rodents during a 60 sec stimulation episode. **A**. Temporal evolution of the cerebral blood flow chosen as an input to the model during a simulated 60 sec stimulation episode *in vivo* (grey area). This specific time course closely matches measurements in rodents during functional forepaw or whisker stimulation [50, 51]. Note that the blood flow only significantly increases approximately 1 sec after the onset of activation (inset). **B**. Relative fluctuations of the intra-parenchymal oxygen concentration as evoked by a 60 sec stimulation episode with the blood flow as in A. These results closely match the experimental results of Ances et al. [50] (their Fig. 1). **C**. Relative fluctuations of tissue lactate (pink line) and of extracellular lactate (black line) during the same 60 sec stimulation episode as in A to B. These results closely match the experimental results of Hu and Wilson [16] (their Fig. 1). **D**. Net transport of lactate between the four compartments of the model during the same 60 sec stimulation episode as in A to C. Like in the *in vitro* case (Fig. 4), lactate is exported by the astrocyte to the extracellular space (thick red line) and imported by the neuron from the extracellular space (thick blue line; net import is negative by convention in this model). A small amount of lactate is exported from the extracellular space to the capillary at baseline and this export increases by 69% after the end of the stimulation (pink line). The thin red line denotes the activity of the lactate dehydrogenase converting pyruvate into lactate in the astrocytic cytosol while the thin blue line denotes the activity of the lactate dehydrogenase converting lactate into pyruvate in the neuronal cytosol (again, the negative sign is a convention)

One of the hallmarks of a successful model is its ability to reproduce and explain empirical observations. We thus now turn to an *in vivo* situation and compare the predictions of the mathematical model we designed in the precedent sections to two experiments carried out in rats. Tissue oxygen and lactate during stimulus (Fig. 5B and C), and lactate transfer between compartments were compared (Fig. 5D).

Upon stimulation, CBF increases after a delay of ~1 sec (see Equation 7), quickly peaks before relaxing to an elevated plateau. This pattern matches neurovascular responses observed in rodents in response to sustained sensory stimulation (for instance by mechanical activation of the whiskers [50, 51] (Fig. 5A)). The 1 sec delay seen in panel A is hardcoded in the model (see Equation 7). This matches our own observations that CBF only starts to increase above its baseline ~0.5–1 sec after the onset of stimulation [51, 59].

Because neuronal activity slightly precedes functional hyperemia, the oxygen concentration initially dips below its resting value before rising as cerebral blood flow finally increases after the 1 sec delay (Fig. 5B inset). The dip in oxygen below baseline levels after stimulation has ceased reflects the rapid decrease in the replenishment rate by blood at a time when oxygen is still consumed to replenish the ATP that is used to fuel the Na, K-ATPase pump.

Extracellular lactate is consumed throughout the stimulation. Its concentration initially dips until the cerebral blood flow increases and leads to a sustained overproduction of lactate (Fig. 5C). At rest, the astrocytic compartment exports lactate to the extracellular space, part of which is taken up by the neuronal compartment for energy production (Fig. 5D), the rest being exported to the circulation. Upon stimulation, export of extracellular lactate to the circulation is reduced while import into neurons is increased. Export of lactate to the extracellular space by the astrocytic compartment is also increased but with a delay and explains the initial dip in concentration. In the recovery period, all transports slowly return back to their baseline values explaining the long lasting overshoot of extracellular and tissue lactate. Export of extracellular lactate to the circulation is durably increased in the recovery period (Fig. 5D; pink line).

Fig. 5 shows results of simulations independent from the simulations that yielded Fig. 2 to 4 where the model was constrained to reproduce the experimental results from ref. [23]. In these new simulations, not only is the temporal course of tissue oxygen qualitatively predicted, but the amplitude of fluctuations is, to some extent, quantitatively predicted as well. Our model predicts that the oxygen pressure first drops by -1.7% (inset), then overshoots at +17.7% before stabilizing 2.4% above its baseline in the last 20 sec of the stimulation. Finally, it undershoots to -2.6% in the post-stimulus period. These figures are to be compared with the values reported by [50], namely, an initial drop at -1.8%, an overshoot at +19.9%, a stabilization 1.7% above the baseline and a final undershoot at -3.1%.

The consumption of lactate closely tracks the stimulation dependent oxygen consumption but lacks the inflections corresponding to CBF changes as it is less affected by the blood flow (Fig. 5D). Lactate is exported by the astrocyte to the extracellular space (thick red line) and imported by the neuron from the extracellular space (thick blue line; net import is negative by convention in this model). A small amount of lactate is exported from the extracellular space to the capillary at baseline and this export increases by 69% after the end of the stimulation (pink line). The thin red line denotes the activity of the lactate dehydrogenase converting pyruvate into lactate in the astrocytic cytosol while the thin blue line denotes the activity of the lactate dehydrogenase converting lactate into pyruvate in the neuronal cytosol (again, the negative sign is a convention). These net transfers all contribute to the evolution of the tissue and extracellular lactate concentrations (Fig. 5C). This prediction closely matches the experimental results of Hu and Wilson [16] (their Fig. 1).

### The biophysical model correctly predicts human glucose and oxygen utilization during brain activation

**Figure 6:**
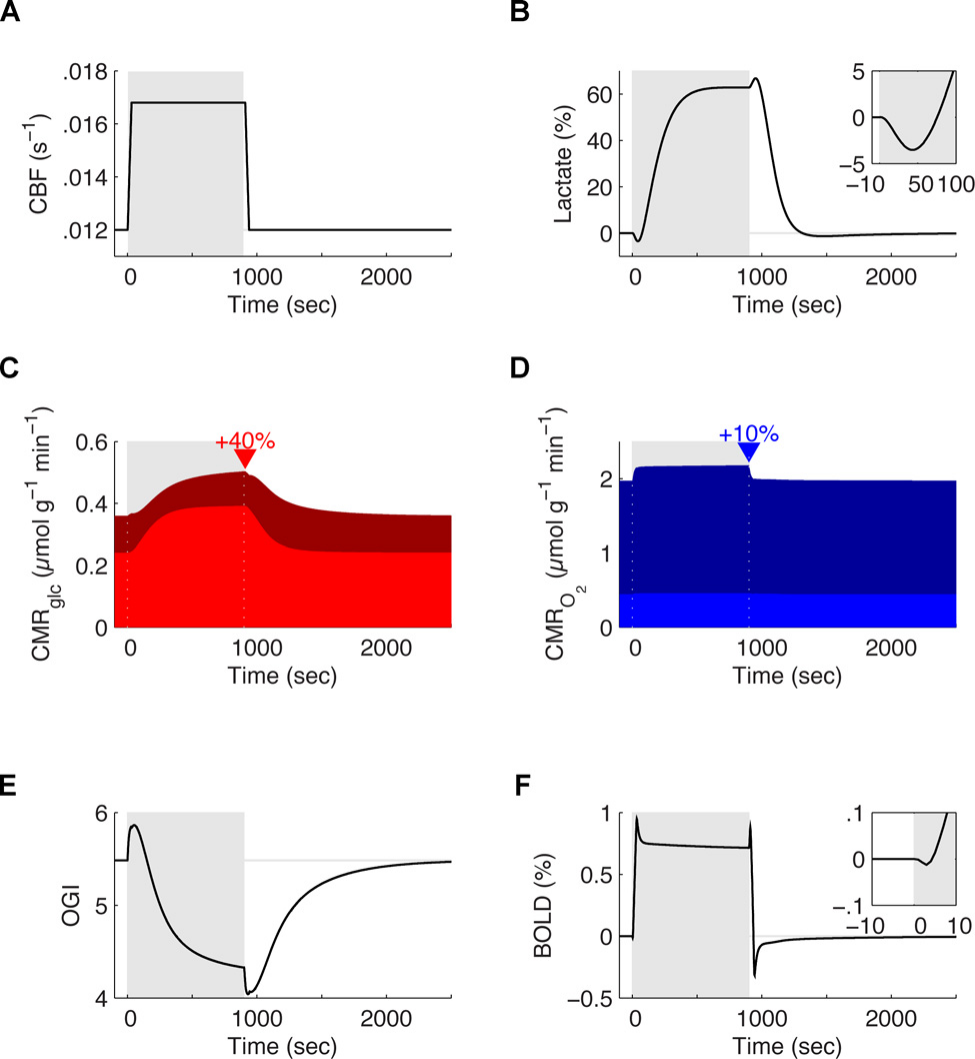
Predicted evoked responses of tissue lactate, CMR_glc_, CMR_O2_, oxygen-glucose index (OGI) and BOLD *in vivo* in humans. **A**. Temporal evolution of the cerebral blood flow chosen as an input to the model during a simulated 900 sec stimulation episode *in vivo* (grey area). This specific time course closely matches *in vivo* measurements in humans during imaging experiments [52, 60]. **B**. Relative fluctuations of tissue lactate concentration during the same stimulation episode as in A. The model predicts an initial lactate dip followed by a 60% increase sustained till the end of the stimulation. The presence of a dip matches experimental data from Mangia et al. [17]. **C and D**. Cerebral metabolic rate of glucose consumption (CMR_glc_) and cerebral metabolic rate of oxygen consumption (CMR_O2_) during the same 900 sec stimulation episode as in A to B. In both cases, the light area corresponds to the contribution of the astrocytic compartment towards the total tissue consumption, while the dark area corresponds to the contribution of the neuronal compartment. While glucose consumption increases by about 40%, the increase is mostly due to the astrocytic compartment (light red) with the neuronal glucose utilization even slightly decreasing at the onset of activation (dark red). On the contrary, while oxygen utilization increases by about 10%, most of this increase is due to the neuronal compartment (dark blue) with the astrocytic oxygen utilization being almost constant (light blue). **E**. The predicted ratio of CMR_O2_ to CMR_glc_ or oxygen-glucose index (OGI) during the same 900 sec stimulation episode as in A to D. **F**. Predicted BOLD signal for the same 900 sec stimulation episode as in A to E. Like the tissue lactate concentration (B), the BOLD shows a clear dip at the onset of activation

We then compared new simulations to measurements from human subjects [26, 52, 60]. As in Fig. 5, the neurovascular response was adapted from experimental measurements (Fig. 6A). Simulated levels of lactate (Fig. 6B), the cerebral metabolic rate for glucose (Fig. 6C), the cerebral metabolic rate for oxygen (Fig. 6D), the ratio of cerebral uptake of O_2_ to cerebral uptake of glucose (Oxygen-Glucose Index or OGI) (Fig. 6E), as well as the blood-oxygen-level dependent signal (Fig. 6F) all supported a validation of the model.

The model qualitatively and to some extent quantitatively predicted well-known features of these various macroscopic observables, including the BOLD signal [52, 60], despite extrapolation from multiple sources. For instance, after an initial dip [17], the tissue lactate concentration reached a plateau that last until the end of the stimulation [61]. The initial dip in extracellular lactate (Fig. 6B inset) is representative of a surge in lactate consumption at the beginning of neural activity, and a symptom of the intrinsic latency in the start-up of the ANLS and of functional hyperaemia. The time lag between the onset and offset of neuronal activation and the onset and offset of CBF also explain other transients such as the slow recovery of the lactate concentration after the cessation of neural activity [61]. Relative increase in glucose and oxygen consumption also matched experimental results and led to a decreased OGI during stimulation [26].

## Discussion

There is mounting evidence in support of a metabolic link between neurons and glial cells. The most prominent experimentally-based conceptual model of neuron-glia metabolic coupling, the astrocyte-neuron lactate shuttle (ANLS), has raised controversies, not so much for the proposed energy-dependent link between the two cell types, but because certain details and mechanisms are debated [11, 62]. Although experimental evidence gathered over the last two decades largely supports it [11], experimentally untangling this system is challenging.

Our detailed biophysical model of the NGV ensemble expands on previous models in four distinct ways. First, a shuttling of lactate from astrocytes to neurons emerged in response to activation. Second, the model is consistent with increased neuronal oxidative metabolism and delayed increased astrocytic glycolysis for generating the activity-dependent NADH transients. Third, the model correctly predicts the dynamics of tissue lactate and oxygen as observed *in vivo* in rats. Fourth, the model correctly predicts with good quantitative precision the temporal dynamics of tissue lactate, CMR_glc_, CMR_O2_ and of the BOLD signal as reported in human studies. These findings not only support the ANLS hypothesis but also provide a quantitative mathematical description of the metabolic activation in neurons and astrocytes, as well as the macroscopic measurements obtained with brain imaging techniques.

The blood oxygen-level dependent (BOLD) signal which forms the basis of the functional magnetic resonance imaging (fMRI) technology reports fluctuations in brain activity, the molecular and cellular mechanisms of which are still incompletely understood [8, 63]. Although somewhat limited in representing detailed processes, our use of the Buxton model was sufficient to correctly predict known features of the BOLD signal (Fig. 6F). Ultimately, modeling efforts that build on our work will have to include more detailed descriptions of blood flow regulation. For instance, blood flow regulation in the brain was recently suggested to happen in the microvasculature at the capillary level by active dilation of pericytes [64]. Subsequent efforts will need to focus on the daunting task of modeling the numerous pathways that relate neuronal activity to functional hyperaemia [65].

Recent modeling of the neuron-astrocyte cross-talk during oscillations linked to blood oxygenation levels verified the possibility that the slow fMRI BOLD signals might reflect the spontaneous ongoing activity of neuroglial networks [66]. Our results support this view by accurately modeling results from human imaging experiments [52, 60]. A course for future modeling will be to examine and model data from multi-modal imaging experiments [67].

As noted in the introduction, controversy surrounds the directionality of lactate flow in the brain with a neuron-to-astrocyte direction proposed by some. Here, following the arguments delineated in Jolivet et al. [42], we imposed that the largest fraction of glucose should go to astrocytes (there is no controversy that neurons are responsible for the vast majority of oxygen consumption). We derive confidence in our model from the fact that it correctly predicts a vast array of *in vivo* experimental findings while being only loosely constrained by *in vitro* experimental findings and by the imposed compartmentalization of glucose uptake between neurons and astrocytes. As argued in Jolivet et al. [42], metabolic shuttling between the astrocytic and neuronal compartments originates from the imbalance between the high oxygen consumption of neurons and their limited glucose utilization for ATP production purposes. Unlike astrocytes, neurons are unable to up-regulate their rate of glycolysis in response to increased activity due to constitutive inhibition of the rate limiting glycolytic enzyme Pfkfb3 (6-phosphofructo-2-kinase/fructose-2, 6-bisphosphatase 3) [68]. This mechanism is crucial to neuronal defenses against reactive oxygen species. Additional *in vitro* mechanisms support that compartmentalization includes the existence of glutamate-induced glycolysis in astrocytes [5, 69], glutamate-induced inhibition of glucose transport in neurons [70] and the involvement of extracellular increases in potassium inducing an Na, K-ATPase-dependent activation of glycolysis in astrocytes [71]. However, it is to be noted that if the proportion of glucose directly taken up by neurons was to be increased, the astrocyte-to-neuron lactate shuttle would be reduced in amplitude, and its direction eventually reversed if neurons were consuming glucose in excess of what they oxidize (see [42] for further discussion of this question).

Metabolic phenotypes have been suggested that convey a metabolic identity depending on how they utilize the various oxidative and non-oxidative pathways [14]. The re-equilibration of the constituents of extracellular space, a process involving physical flushing mediated by astrocytes, is also now thought to be one of the key housekeeping functions of sleep [72] and the disruption of normal metabolic processes is suggested to underlie the progression of neurodegenerative diseases such as Alzheimer’s [73]. Lactate, whether sourced from glia or plasma, is associated with neuroprotection [32–34]. Assuming the ANLS is indeed taking place, we are left to question: What is it good for?

Neurons do not appear to suffer functional consequences as a result of their metabolic peculiarities. Lactate can sustain prolonged firing in neurons more efficiently than glucose in culture and can preferentially support activity in both resting and active states *in vitro* and *in vivo* [30, 31, 74]. Recently, it was shown in the subfornical organ that this pathway can also affect the dynamics of the local neural network by modulating the excitability of GABAergic neurons through the regulation of ATP-dependent potassium channels [36]. It is not necessary perhaps to preclude the use of glucose by both neurons and glia under certain circumstances as has been suggested by a computational model studying the ATP supply to neurons under hypoxic conditions [75], and as is indeed suggested by our own results (see Fig. 4B, 5D and 6C). Finally, extracellular lactate might also act as a messenger to the vasculature [28] and it is thus possible that the ANLS plays a role as one of the pathways regulating functional hyperaemia.

Astrocytes also support memory formation by supplying neurons with lactate [76]. So central is the ANLS to the normal function of the brain that learning, as measured by LTP and long term memory formation in the hippocampus of rats, is abolished by interfering with the transport of lactate from astrocytes to neurons [76]. Consistent with those findings, lactate, but not glucose, has been show to induce the expression of plasticity genes such as Arc, Zif 268 and BDNF *in vitro* in neurons and *in vivo* [77]. Further, the mechanism by which glucose enhances memory storage has been shown to involve the neuronal consumption of lactate [78].

### Summary

We present here the first temporal multi-scale model of the NGV that accurately reflects experimental observations in multiple settings and organisms. These findings not only support the ANLS hypothesis but also provide a quantitative mathematical description of the metabolic activation in neurons and astrocytes, as well as of the macroscopic measurements obtained with functional brain imaging techniques.
